# Phase II evaluation of copanlisib, a selective inhibitor of Pi3kca, in patients with persistent or recurrent endometrial carcinoma harboring PIK3CA hotspot mutations: An NRG Oncology study (NRG-GY008)

**DOI:** 10.1016/j.gore.2019.100532

**Published:** 2020-01-02

**Authors:** Alessandro D. Santin, Virginia Filiaci, Stefania Bellone, Elena S. Ratner, Cara A. Mathews, Guilherme Cantuaria, Camille C. Gunderson, Teresa Rutledge, Barbara M. Buttin, Heather A. Lankes, Michael Frumovitz, Samir N. Khleif, Warner K. Huh, Michael J. Birrer

**Affiliations:** a Department of Obstetrics, Gynecology & Reproductive Services, Yale University School of Medicine, New Haven, CT 06520, USA; b NRG Oncology Statistical and Data Management Center, Roswell Park Comprehensive Cancer Center, Buffalo, NY 14263, USA; c Medical Oncology, Women & Infants Hospital, 101 Dudley Street, Providence, RI 02905, USA; d Gynecologic Oncology, 980 Johnson Ferry Road #910, Atlanta, GA 30342, USA; e Department of Obstetrics & Gynecology, University of Oklahoma, The Stephenson Cancer Center, 800 NE 10^th^ Street, Suite 2500, Oklahoma City, OK 73104, USA; f Gynecologic Oncology, University of New Mexico, 1201 Camino de Salud, Albuquerque, NM 87102, USA; g Department of Obstetrics & Gynecology, Northwestern Medicine Regional Medical Group, 4405 Weaver Parkway, Warrenville, IL 60555-3269, USA; h NRG Oncology, Operations Center-Philadelphia East, Philadelphia, PA, USA; i Division of Gynecologic Oncology, Department of Obstetrics and Gynecology, The Ohio State University Wexner Medical Center, Columbus, OH, USA; j Department of Gynecologic Oncology, The University of Texas MD Anderson Cancer Center, 1515 Holcombe Blvd, Houston, TX 77030, USA; k Director, The Loop Immuno-Oncology Research Laboratory, Lombardi Cancer Center, Georgetown University, Washington, DC 20057, USA; l Division of Gynecologic Oncology, University of Alabama at Birmingham, Birmingham AL 35205, USA; m Division of Hematology/Oncology, O’Neal Cancer Center University of Alabama, 176F 10390, 619 19^th^ Street S, Birmingham, AL, USA

**Keywords:** Endometrial neoplasms, Copanlisib, Pik3ca, Targeted treatment

## Abstract

**Purpose:**

NRG Oncology conducted a phase II trial to assess the antitumor activity and tolerability of copanlisib, a selective inhibitor of PIK3CA, in persistent or recurrent endometrial carcinoma harboring hotspot PIK3CA mutations.

**Patients and methods:**

Eligible patients had endometrial cancer with endometrioid, serous or mixed histology, a somatic PIK3CA gene mutation, measurable disease, and GOG performance status ≤2. Treatment consisted of IV copanlisib (60 mg weekly, day 1, 8 and 15 of 28-day cycle) until disease progression or prohibitive toxicity. The primary endpoints of the study were objective tumor response as assessed by RECIST 1.1 and to determine the nature and degree of toxicity of copanlisib as assessed by CTCAE version 4. The study used a 2-stage group sequential design.

**Results:**

Eleven patients were enrolled onto stage I of the treatment trial. Five patients had endometrioid, four serous and two had a tumor of mixed histology. The most common PIK3CA mutation was Q546X (n = 3) in exon 9. The most common grade 3 or 4 AE was hyperglycemia. No grade 5 adverse events were reported. No clinical responses were detected. Six patients had a best overall response of stable disease. Of 11 who initiated treatment, 10 progressed on treatment. One patient with stable disease on copanlisib withdrew from treatment secondary to relocation. The median progression-free survival (PFS) was 2.8 months; at 6 months 27% were alive, progression-free. The median overall survival (OS) was 15.2 months. Due to the lack of CR/PR continuation of accrual to the second stage of accrual was not warranted.

**Conclusion:**

Copanlisib is well tolerated but has limited activity as a single agent in this population.

## Highlights

• Endometrial cancer commonly harbors hotspot mutations in the PIK3CA gene.
• NRG-GY008 evaluated the activity of copanlisib, an inhibitor of PIK3CA, in recurrent endometrial cancer patients.
• Copanlisib has an acceptable safety profile but low antitumor activity in endometrial cancer.
• Combinations of copanlisib may be necessary to increase clinical responses in endometrial cancer patients.

## 1 Introduction

Endometrial cancer (EC) is the most common gynecologic malignancy with 63,230 new cases and 11,350 estimated deaths related to the disease in the United States in 2018 (Siegel et al., 2018). ECs have historically been designated as Type I or Type II (Bokhman, 1983). Type I endometrial cancer accounts for 65–70% of cases and is associated with grade 1–2 endometrioid histology, younger age of onset, retention of estrogen receptor (ER) and progesterone receptor (PR) status, a history of unopposed estrogen, and deletions in k-Ras, PTEN, or mismatch repair mechanisms (Bokhman, 1983, Matthews et al., 1997). In contrast, Type II endometrial cancer is associated with serous, clear cell or grade 3 endometrioid histology, loss of ER/PR, black race, absence of unopposed estrogen, presentation at later stage, reduced E-cadherin expression, aneuploidy, mutations in p53 and HER2/Neu overexpression (Bokhman, 1983, Matthews et al., 1997, Kandoth et al., 2013, Zhao et al., 2013). Type II endometrial cancer is typically more aggressive than type I cancer and has a poorer prognosis.

Recently, using an integrated genomic, epigenomic, transcriptomic and proteomic approach, The Cancer Genome Atlas (TCGA) Research Network provided compelling evidence that endometrial cancers result from heterogeneous somatic mutations and, accordingly, classified endometrial cancers into four categories: (1) polymerase epsilon (POLE)-ultra-mutated, (2) microsatellite instability hyper-mutated, (3) copy-number low and (4) copy-number high, serous-like (Kandoth et al., 2013). The genetic aberrations of endometrial carcinomas may therefore represent a novel tool to classify these tumors and guide adjuvant treatment in women with recurrent chemotherapy-resistant disease.

The phosphatidylinositol-3-kinase (PI3KCA) gene encodes for a heterodimeric protein with an 85-kDa regulatory subunit (PI3KR1) and a 110-kDa catalytic subunit (PI3KCA) (Vanhaesebroeck et al., 2010). PI3K phosphorylates a series of membrane phospholipids, catalyzing the transfer of ATP (adenosine triphosphate)-derived phosphate, thereby forming secondary messenger lipids phosphatidylinositol 3,4-bisphosphate and phosphatidylinositol 3,4,5-trisphosphate and initiating the downstream AKT/mTOR signaling cascade that regulates cell growth (Vanhaesebroeck et al., 2010). The central role of PI3K activation in tumor cell biology has prompted an effort to target PI3K and/or downstream kinases such as AKT and mammalian target of rapamycin (mTOR) in endometrial cancers where mutations in PIK3CA have been reported at high frequency in both Type I and Type II tumors (Pavlidou and Vlahos, 2014). Accordingly, several groups have recently reported the activity of multiple PI3KCA and mTOR inhibitors in endometrial cancer in preclinical studies. For example, apitolisib (GDC-0980, Genentech, South San Francisco, CA), a potent inhibitor of class I PI3K and mTOR kinase (TORC1/2), has shown significant activity *in vitro* and *in vivo* against biologically aggressive endometrial tumors harboring PI3K driver mutations (English et al., 2013). Furthermore, AZD8055, a dual mTORC1/2 inhibitor, demonstrated significant tumor growth inhibition in high HER-2/neu-expressor endometrial cancers *in vitro* (English, et al., 2013) as well as *in vivo* regression in breast, lung, colon, prostate, and uterine xenograft models (Chresta et al., 2010). Taselisib, GDC-0032 (Genentech, South San Francisco, CA), a novel, oral, selective inhibitor of PI3KCA, has been shown to be active in uterine serous carcinoma (USC) mouse xenografts harboring PI3KCA mutations and overexpressing HER2/neu (Lopez et al., 2014).

Copanlisib (BAY 80-6946) is an intravenous, potent and highly selective pan-Class I PI3K inhibitor with predominant activity towards the PI3Kα and PI3Kδ isoform showing superior antitumor activity (>40-fold) in PI3KCA mutant tumors in preclinical studies and promising clinical activity and manageable toxicity in Phase I and II clinical trials (Liu et al., 2013, Anonymous, 2014, Patnaik et al., 2016, Dreyling et al., 2017). Copanlisib (ALIQOPA, Bayer HealthCare Pharmaceuticals Inc.) has been recently approved by the Food and Drug Administration (FDA) for the treatment of adult patients with relapsed follicular lymphoma who have received at least two prior systemic therapies. Most common side effects of copanlisib in phase I/II clinical studies included hyperglycemia and transient Grade 3 hypertension. Copanlisib not only inhibits PI3Kα with IC50 of 0.5 nM, but also PI3Kδ with IC50 of 0.7 nM. In vivo, single intravenous administration of copanlisib exhibited higher exposure and prolonged inhibition of pAKT levels in tumors versus plasma. Copanlisib may represent a promising agent with differential pharmacologic and pharmacokinetic properties for the treatment of PI3K-dependent human tumors.

NRG Oncology conducted a phase II trial of single-agent copanlisib in patients with persistent or recurrent endometrial carcinoma harboring PI3KCA mutations. The primary endpoint of this study was the frequency of patients with tumor responses. The secondary objectives were to estimate the 6-month progression-free survival (PFS) and median PFS, the distribution of overall survival (OS), the duration of objective response, and the frequency and severity of adverse events.

## 2 Materials and methods

### 2.1 Eligibility

Eligibility criteria included patients with persistent or recurrent endometrial cancer (endometrioid adenocarcinoma, serous adenocarcinoma, undifferentiated carcinoma, mixed epithelial carcinoma or adenocarcinoma not otherwise specified) that was measurable by Response Evaluation Criteria in Solid Tumors (RECIST); treated with at least one prior cytotoxic regimen; with somatic “hotspots” PIK3CA gene mutation (i.e., R88Q in exon 1, N345K in exon 4, C420R in exon 7, E542K, E545X [E545A, E545D, E545G, and E545K], Q546X [Q546E, Q546K, Q546L, and Q546R] in exon 9, and M1043I, H1047X [H1047L, H1047R, and H1047Y], or G1049R in exon 20) in a representative primary or metastatic archival tumor sample by the Roche COBAS® PIK3CA Mutation Test at Q2 Solutions (Marietta, GA, USA); GOG performance status (PS) of no worse than 2; and adequate hematologic (absolute neutrophil count ≥1500/µL and platelets ≥100,000/µL), renal (serum creatinine ≤1.5× the institutional upper limit of normal [ULN]); hepatic (serum bilirubin ≤1.5× ULN, and both AST and alkaline phosphatase ≤2.5× ULN) laboratory values. Histologic documentation of the original primary tumor was required with a pathology report.

Patients with other malignancies evident within 5 years, prior non-cytotoxic therapy with any PI3K/AKT/mTor pathway inhibitor, infection requiring antibiotics, active bleeding or central nervous system (CNS) disease (craniospinal metastases, uncontrolled seizure disorder) were ineligible. Patients were also excluded for significant cardiovascular disease (uncontrolled hypertension, unstable angina, uncontrolled congestive heart failure, or uncontrolled arrhythmias within 6 months of registration), pregnancy or nursing, or major surgical procedures within 30 days or anticipated while on study. Diabetic patients (Type I or II diabetes mellitus) must have had baseline HbA1c levels not higher than 8.5% at study entry. Patients with hypertension on medical management must have had systolic blood pressure <150 mmHG or diastolic pressure <90 mmHG at study entry. The study received local institutional review board approval at participating institutions and all patients provided authorization permitting the release of personal health information and gave informed consent according to institutional and federal guidelines before enrollment.

### 2.2 Treatment

Patients were treated with IV copanlisib (60 mg over 1 h weekly, day 1, 8 and 15 of 28-day cycle) until disease progression or prohibitive toxicity. Each 28 day period was considered 1 cycle. Toxicity was monitored with history, physical examination, and laboratory assessment before each treatment cycle, with adverse events defined and graded according to National Cancer Institute Common Terminology Criteria version 4.0.

### 2.3 Evaluations

Activity of copanlisib was assessed according to RECIST (version 1.1), either by computed tomography or magnetic resonance imaging at baseline, and before every other cycle (regardless of delays and/or changes in treatment schedule) for the measurement of target lesions, the classification of clinical response, and the determination of disease progression. Therapy was discontinued if there was disease progression, unacceptable toxicity, receipt of other anticancer therapy, or voluntary withdrawal.

### 2.4 Statistical design

A flexible, minimax 2-stage design (Chen and Ng, 1998) was chosen for this study to test the null hypothesis that the proportion responding is no greater than 5% against the alternative that the proportion responding is at least 25%. The design has an average expected sample size of 18.5 and a probability of early termination of 55%. These average probabilities are computed from the individual probabilities averaged over all permitted accrual combinations and assuming each combination is equally likely. Between 10 and 17 patients (target of 15) were to be enrolled onto the treatment component in the first stage of accrual. If the number responding is less than or equal to 0/(10–16) or 1/17, then the study would terminate early and the regimen declared uninteresting. Otherwise, with medical judgment indicating, the study will accrue to a second stage with a cumulative sample size between 21 and 28 (target of 25). The design described has 90% statistical power under the alternative specified. Type I error is set at 0.05 (one-sided hypothesis test). All enrolled patients with recurrent endometrial tumors that are shown to harbor eligible PIK3CA mutations and that initiate study treatment will be included in the analysis of objective response. Exact 95% confidence limits are reported for the response proportion estimate. The frequency and severity of adverse events (AE) is tabulated for patients by the highest grade of overall adverse events, by highest AE grade within each system organ class, and by highest grade for each adverse event term observed, regardless attribution. The Kaplan Meier estimation method was used for progression-free and overall survival.

## 3 Results

### 3.1 Patient and disease characteristics

This study was opened to accrual on September 16, 2016. Screening for PIK3CA mutations by the cobas® PIK3CA mutation test performed by Q2 Solutions was initiated on January 23, 2017. Through April 13, 2017, 42 patients were registered for screening and, through August 7, 2017, 11 patients were found to harbor “hot spots” PIK3CA mutations and were enrolled in the trial. The patient and disease characteristics including previous treatments for all eligible and evaluable patients are summarized in Table 1. The median age was 68 years (Table 1). All patients on treatment were of non-Hispanic or Latino ethnicity. All but one self-declared to be Caucasian. Ten patients enrolled with a performance status of no worse than 1. Five patients had endometrioid tumors, four had serous tumors and two had a tumor of mixed histology. The most common mutation identified was Q546X (n = 3) in exon 9 (Table 2).

**Table 1:** Patient and tumor characteristics for all eligible patients.

Characteristic	Frequency	Percent
Age (years)		
50–59	3	27.3
60–69	4	36.4
70–79	4	36.4
Ethnicity		
Not Hispanic or Latino	11	100.0
Race		
Asian	1	9.1
White	10	90.9
Performance Status		
0	5	45.5
1	5	45.5
2	1	9.1
Histology		
Endometrioid adenocarcinoma, FIGO grade 2	2	18.2
Endometrioid adenocarcinoma, FIGO grade 3	3	27.3
Mixed epithelial carcinoma	2	18.2
Serous adenocarcinoma	4	36.4
Number of prior chemotherapy regimens		
1	6	54.5
2	1	9.1
3	3	27.3
5	1	9.1

**Total**	11	100.0

**Table 2:** Mutation data and response.

Mutation	Histology	Best response	N	%
Q546Xt	Endometrioid	Stable	1	9.1
M1043It	Endometrioid	Stable	1	9.1
Q546Xt	Endometrioid	Progression	1	9.1
H1047Xt	Endometrioid	Progression	1	9.1
E545Xt	Endometrioid	Progression	1	9.1
N345Kt	Mixed	Stable	1	9.1
H1047Xt	Mixed	Progression	1	9.1
Q546Xt	Serous	Stable	1	9.1
E542Kt	Serous	Stable	2	18.2
E545Xt	Serous	Progression	1	9.1

### 3.2 Response to treatment

**Fig. 1:**
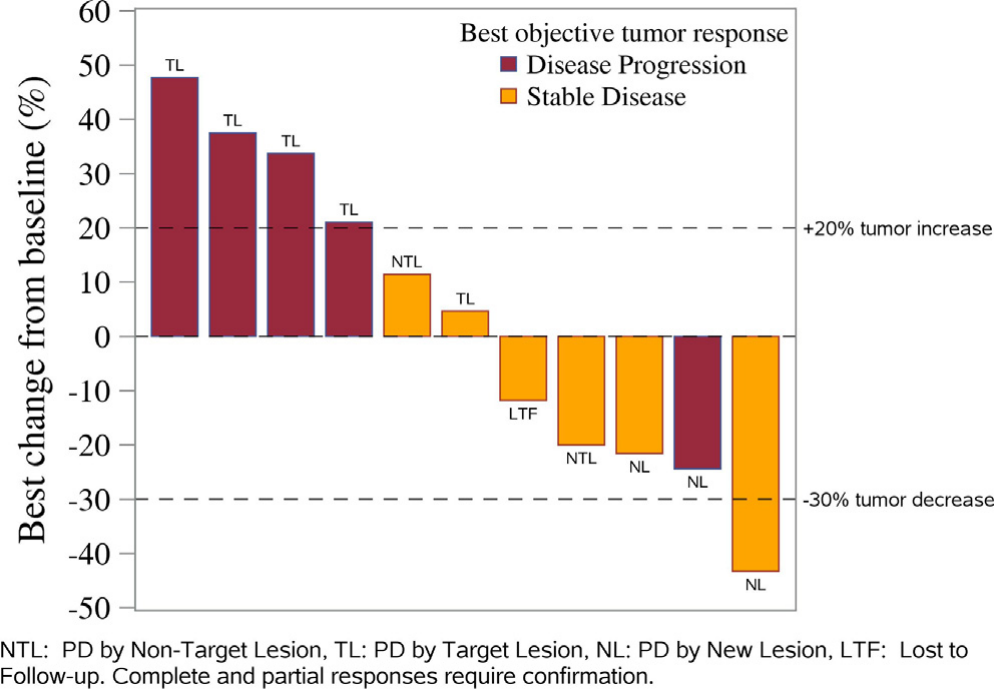
Waterfall plot showing distribution of the best percentage change in target lesion size from baseline for an individual patient. The lines (–30 and +20%) indicate the region with change from baseline that typically represent SD based on RECIST guidelines. Please note that patient with a >30% decrease in tumor volume had a new lesion at the time of the unconfirmed PR. Thus, the patient progressed and a partial response was not confirmed.

**Fig. 2:**
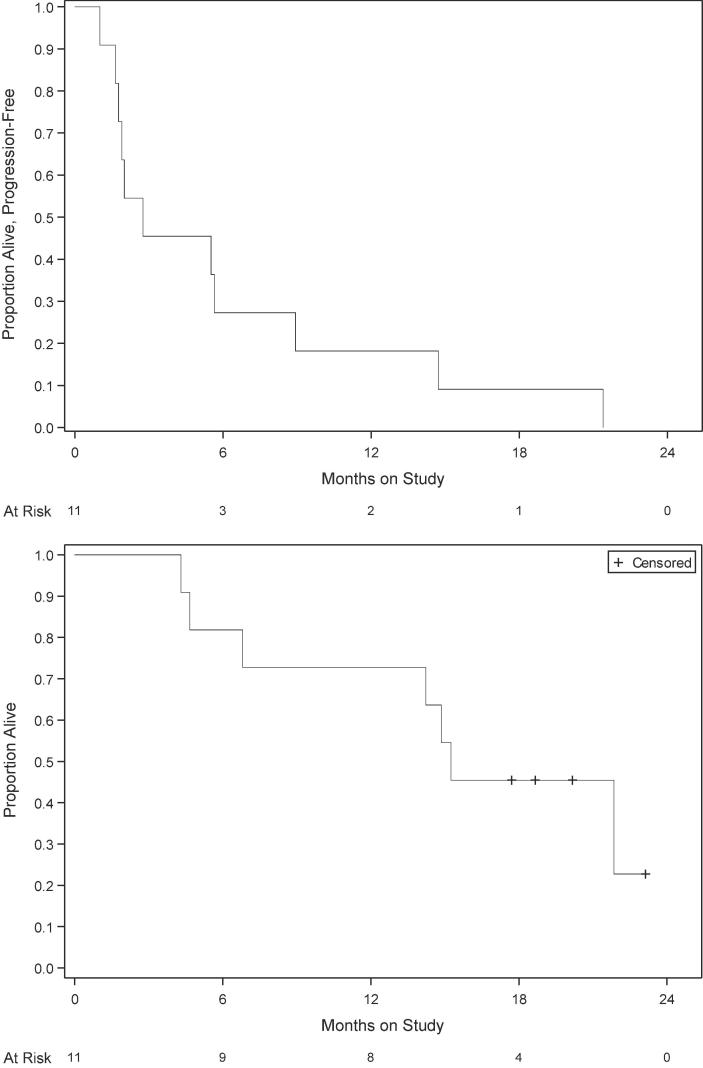
Kaplan-Meier estimates of PFS and OS for the study population of patients treated with copanlisib.

One patient withdrew from study treatment after initiating treatment (due to relocation in another state) with stable disease and 10 patients discontinued treatment due to disease progression. Six patients were on treatment for 3 cycles or less. There were no confirmed complete or partial responses reported at the time of analysis. Six patients had a best overall response of stable disease. Five patients demonstrated decrease in the diameter of target lesions during treatment while 5 had increased disease as best response (waterfall plot, Fig. 1 and Table 3). Two patients with decreases in the sum of dimensions had co-incident new lesions identified. Continuation of accrual to the second stage of accrual was not warranted. The median progression-free survival (PFS) is 2.8 months (Fig. 2). With a median follow-up of 20 months, the median overall survival is 15.2 months. Seven of the 11 patients have died.

**Table 3:** Treatment outcomes.

	Frequency	Percent
Reason off treatment		
Disease progression	10	90.1
Patient withdrew after starting therapy	1	9.1
Best Response		
Disease Progression within 8 weeks	5	45.5
Stable Disease lasting at least 8 weeks	6	54.5

### 3.3 Adverse events

Adverse events are assessed by the NCI Common Terminology Criteria for Adverse events (CTCAE) version 4.0. Eleven participants have received treatment on study. Table 4 summarizes the distribution of patients by the highest grade of overall adverse events and by highest grade within each system organ class of adverse events, regardless attribution. Overall, 7 participants experienced a grade 3 adverse event as their highest reported grade and 2 participants experienced at least one grade 4 AE. The most common grade 3 or 4 AE was hyperglycemia. No grade 5 adverse events have been reported. All reported adverse event terms that had at least one grade 1 or higher adverse event are included in Table 4 within their system organ class.

**Table 4:** Distribution of GY008 patients by highest grade adverse event regardless to attribution by system organ class.

Frequency (%) of patients by grade					
System Organ Class	1	2	3	4	5
Overall Highest Grade	1	1	7	2	0
	(9.1)	(9.1)	(63.6)	(18.2)	(0.0)
Blood and Lymphatic System Disorders	0	2	1	0	0
	(0.0)	(18.2)	(9.1)	(0.0)	(0.0)
Gastrointestinal Disorders	6	5	0	0	0
	(54.5)	(45.5)	(0.0)	(0.0)	(0.0)
General Disorders and Administration Site Conditions	6	0	2	0	0
	(54.5)	(0.0)	(18.2)	(0.0)	(0.0)
Infections and Infestations	0	1	1	0	0
	(0.0)	(9.1)	(9.1)	(0.0)	(0.0)
Investigations	2	2	0	1	0
	(18.2)	(18.2)	(0.0)	(9.1)	(0.0)
Metabolism and Nutrition Disorders	2	0	4	1	0
	(18.2)	(0.0)	(36.4)	(9.1)	(0.0)
Musculoskeletal and Connective Tissue Disorders	2	2	0	0	0
	(18.2)	(18.2)	(0.0)	(0.0)	(0.0)
Nervous System Disorders	0	2	2	0	0
	(0.0)	(18.2)	(18.2)	(0.0)	(0.0)
Psychiatric Disorders	2	0	0	0	0
	(18.2)	(0.0)	(0.0)	(0.0)	(0.0)
Renal and Urinary Disorders	3	0	0	0	0
	(27.3)	(0.0)	(0.0)	(0.0)	(0.0)
Reproductive System and Breast Disorders	1	0	0	0	0
	(9.1)	(0.0)	(0.0)	(0.0)	(0.0)
Respiratory, Thoracic and Mediastinal Disorders	2	1	0	0	0
	(18.2)	(9.1)	(0.0)	(0.0)	(0.0)
Skin and Subcutaneous Tissue Disorders	2	2	1	0	0
	(18.2)	(18.2)	(9.1)	(0.0)	(0.0)
Vascular Disorders	1	1	0	0	0
	(9.1)	(9.1)	(0.0)	(0.0)	(0.0)

## 4 Discussion

Patients with recurrent, chemotherapy-resistant endometrial cancer have a poor prognosis. The GOG has screened over twenty agents for activity in the treatment of persistent or recurrent endometrial tumors with response rates ranging between 0 and 27%. Only two of these agents (i.e., paclitaxel, bevacizumab) produced response rates in excess of 15%. The development of innovative, effective therapies against recurrent, chemotherapy-resistant endometrial cancer remains a high priority.

Comprehensive genetic landscape studies from the TCGA network, our lab and others have recently identified multiple deranged genes/pathways with aberrant activation in a large proportion of both in Type I and Type II endometrial tumors (Kandoth et al., 2013, Zhao et al., 2013). PIK3CA was identified as one of the most commonly mutated “driver genes” in endometrial tumors and, accordingly, multiple PI3KCA, AKT and mTOR targeted inhibitors are currently in Phase I-III clinical trials against solid tumors including endometrial cancer.

Copanlisib (BAY 80-6946) is an intravenous, highly selective pan-Class I PI3K inhibitor showing superior antitumor activity (>40-fold) in PI3KCA mutant tumors in preclinical studies and promising clinical activity and manageable toxicity in Phase I and II clinical trials (Liu et al., 2013, Anonymous, 2014, Patnaik et al., 2016, Dreyling et al., 2017). Importantly, successful clinical trials in patients with lymphoma have recently granted copanlisib approval by the Food and Drug Administration (FDA) for relapsed follicular lymphoma. Approval was based on efficacy results in 104 patients with relapsed follicular lymphoma enrolled in an open-label, single-arm, multicenter, phase 2 trial (Dreyling et al., 2017 Sep 1). The complete response rate was 14.4% and partial response rate was 44.2%. The safety population included 168 patients with follicular lymphoma and other hematologic malignancies treated with the recommended copanlisib dosing regimen. In this registration study, common adverse reactions in greater than 20% of patients included hyperglycemia, diarrhea, fatigue, hypertension, fever, leukopenia, neutropenia, nausea, lower respiratory tract infections, transaminitis and thrombocytopenia. The most common grade 3–4 adverse reactions included hyperglycemia, leukopenia, hypertension, neutropenia, and lower respiratory tract infections. Serious non-infectious pneumonitis occurred in 6% of patients.

The current study was designed by NRG to estimate the therapeutic activity of single-agent copanlisib in the setting of treatment failure with one or more prior regimens for metastatic/recurrent endometrial cancer harboring hotspot PIK3CA mutations. In this group of pretreated patients copanlisib showed limited activity, with a response rate of 0% (confirmed partial or complete response, 95% confidence interval of 0–28.5%) and 6 (54%) of patients demonstrating stable disease as best response. Three patients remained without progression after 6 months on protocol. Because the required number of patients with responses to declare the regimen interesting was not reached at first stage, continuation of accrual to the second stage was not warranted. A median OS of 15.2 months was found in the current study. It is worth noting, however, that 4 out of 11 of the GY008 study patients (36%), although not reaching the 30% RECIST 1.1 criteria cut-off for a PR, demonstrated decrease in the sum of the diameters of the target lesions during treatment in the absence of new lesions, and 27% of patients survived progression free for at least 6 months. Stable disease was seen in all three histologic groups including 3 with serous histology.

The safety profile of copanlisib has been studied in previous reports (Liu et al., 2013, Anonymous, 2014, Patnaik et al., 2016, Dreyling et al., 2017). The present study did not identify any new toxicities or an increased frequency of currently reported toxicities of copanlisib in endometrial cancer patients with persistent or recurrent disease. The most common Grade 3 and 4 adverse events reported in the study were related to hyperglycemia. No grade 5 adverse event have been reported.

In the current trial, patients were prescreened on the basis of PIK3CA “hotspot” mutations as detected by the Roche COBAS® PIK3CA Mutation Test at Q2 Solutions (Marietta, GA, USA). Importantly, in endometrial cancer multiple studies have shown that unlike other human tumors, PI3KCA mutations are distributed throughout the gene. Consistent with this view, Rudd et al., (Rudd et al., 2011) reported that half (29 of 58) of non-synonymous PIK3CA mutations in Type I and Type II cancers are located in exons 1–7 while half are in exons 9 and 20 (i.e., known hotspot mutations). The exons 1–7 mutations have been shown to localize to the ABD, ABD-RBD linker and C2 domains of p110α and similarly to the hot spot mutations located in exon 9 and 20, may increase the levels of phospho-AKTSer473 compared to wild-type p110α (Rudd et al., 2011). Taken together, these preclinical data suggested that in endometrial cancer patients PI3KCA mutations located outside exons 9 and 20 may also represent potential targets for selective pan-Class I PI3K inhibitors such as copanlisib (BAY 80-6946) and accordingly, patients harboring PIK3CA mutation in exons different from 9 and 20 were also eligible for enrollment in the GY008 trial.

While characterization of many of the genetic alterations identified as tumor drivers has led to rational development of highly targeted inhibitors that have greatly improved treatment for some cancer patients, the majority of solid tumors either do not respond or rapidly develop compensatory mechanisms that drive resistance, causing recurrence and death. Our results with copanlisib in PIK3CA mutated recurrent endometrial cancer patients are consistent with the recent literature and suggest limited activity of single agent PIK3CA inhibitors in the treatment of chemotherapy-resistant, recurrent human solid tumors (Janku et al., 2018). Consistent with these clinical data, we recently demonstrated that PIK3CA inhibitors (copanlisib and taselisib) and pan-c-erb-inhibitors (afatinib and neratinib), when used as single agents, while initially highly active *in vivo* against biologically aggressive endometrial cancer models, may rapidly induce the development of compensatory mechanisms of resistance including upregulation of phosphorylated (p)HER2/neu and pEGFR in PIK3CAi-treated/resistant tumors or upregulation of pAKT in neratinib-treated/resistant tumors (Lopez et al., 2014, Lopez et al., 2015). On the basis of these experimental results it has been hypothesized that a double blockade of the HER2/PIK3CA/AKT/mTOR pathway with the combination of neratinib, a pan c-erb inhibitor (to prevent compensatory upregulation of phosphorylated (p)HER2/neu and pEGFR) and taselisib, a PIK3CA inhibitor (to prevent downstream upregulation of pAKT in neratinib-treated/resistant tumors) may represent a more effective approach to prevent or significantly delay the development of tumor resistance in endometrial cancer patients. In agreement with this hypothesis, we found PIK3CA and pan-c-erb inhibitors to be highly synergistic, well-tolerated *in vivo* in animals and able to prevent development of resistance in all preclinical endometrial cancer model so far tested (Lopez et al., 2014, Lopez et al., 2015). More importantly, dual-inhibition therapy initiated after tumor progression in single agent-treated mice was also remarkably effective at inducing tumor regression in large endometrial xenografts resistant to either PIK3CA or pan-ErbB inhibitors (Lopez et al., 2014, Lopez et al., 2015). These preclinical results in relevant preclinical endometrial cancer models are in agreement with recent clinical data in a variety of human tumors, suggesting that combination regimens using highly targeted drugs may improve responses and provide longer clinical benefits to patients, since co-administering of drugs that work by different molecular mechanisms may increase tumor cell killing while reducing the likelihood of drug resistance and minimizing overlapping toxicity. Taken together, these encouraging preclinical data in combination with the limited activity of single agent copanlisib demonstrated in NRG-GY008, support the view that future trial with copanlisib may require use of synergistic combinations in order to induce clinical responses in patients with endometrial cancer.

This study has some limitations within which our findings must be interpreted with caution. First, the clinical data presented derive from the treatment of a small number of recurrent endometrial cancer patients (ie, a total of 11 patients). Second, the study was not designed to perform a comprehensive biomarker analysis of the tumor samples to better understand the level of PI3K pathway activation. This point is noteworthy since a previous report with copanlisib demonstrated a complete clinical responses in an endometrial cancer patient harbroing PIK3CA mutations in combination with PTEN loss (Patnaik et al., 2016).

In conclusion, GY008 study indicates that single agent copanlisib has limited activity in patients with persistent or recurrent endometrial cancer harboring hotspot PIK3CA mutations. Copanlisib was well tolerated in this patient population. In light of the likely weak driver oncogenic activity of mutant PI3K and the rapid upregulation of compensatory feedback mechanisms when PI3K is blocked (Hanker et al., 2019), future trials evaluating the activity of copanlisib in combination with other therapeutics seem warranted.

## CRediT authorship contribution statement

**Alessandro D. Santin:** Conceptualization, Data curation, Investigation, Formal analysis, Writing - original draft, Writing - review & editing. **Virginia Filiaci:** Conceptualization, Data curation, Investigation, Formal analysis, Writing - review & editing. **Stefania Bellone:** Conceptualization, Data curation, Investigation, Formal analysis, Writing - review & editing. **Elena S. Ratner:** Data curation, Investigation, Formal analysis, Writing - review & editing. **Cara A. Mathews:** Data curation, Investigation, Formal analysis, Writing - review & editing. **Guilherme Cantuaria:** Data curation, Investigation, Formal analysis, Writing - review & editing. **Camille C. Gunderson:** Data curation, Investigation, Formal analysis, Writing - review & editing. **Teresa Rutledge:** Data curation, Investigation, Formal analysis, Writing - review & editing. **Barbara M. Buttin:** Data curation, Investigation, Formal analysis, Writing - review & editing. **Heather A. Lankes:** Data curation, Investigation, Formal analysis, Writing - review & editing. **Michael Frumovitz:** Data curation, Investigation, Writing - review & editing. **Samir N. Khleif:** Data curation, Investigation, Writing - review & editing. **Warner K. Huh:** Data curation, Investigation, Writing - review & editing. **Michael J. Birrer:** Data curation, Investigation, Formal analysis, Writing - review & editing.

## Declaration of Competing Interest

Dr. Santin received research grants from Puma, Immunomedics, Gilead, Synthon, Merck, Boehringer-Ingelheim, Genentech and Tesaro and consulting fees from Merck and Tesaro.

Dr. Filiaci reports grants from NIH, during the conduct of the study as well as grants from the GOG Foundation, outside of the submitted work. Dr. O’Cearbhaill reports personal fees from Clovis, personal fees from Tesaro, personal fees from GlaxoSmithKline, outside of the submitted work. Dr. Ratner reports other support received for serving on the Tesaro Advisory Board as well as the Genentech Advisory board which she wishes to disclose. Dr. Mathews reports grants from National Cancer Institute, during the conduct of the study. Dr. Gunderson reports other from Clovis, other from Cordgenics, other from Agenus, outside of the submitted work. All other co-authors have no conflicts of interest to disclose.
