# Corrigendum to “Phase II evaluation of copanlisib, a selective inhibitor of Pi3kca, in patients with persistent or recurrent endometrial carcinoma harboring PIK3CA hotspot mutations: An NRG oncology study (NRG-GY008)” [Gynecol. Oncol. Rep. 31 (2020) 100532]

**DOI:** 10.1016/j.gore.2020.100590

**Published:** 2020-05-28

**Authors:** Alessandro D. Santin, Virginia Filiaci, Stefania Bellone, Roisin O'Cearbhaill, Elena S. Ratner, Cara A. Mathews, Guilherme Cantuaria, Camille C. Gunderson, Teresa Rutledge, Barbara M. Buttin, Heather A. Lankes, Michael J. Birrer

**Affiliations:** a Department of Obstetrics, Gynecology & Reproductive Services, Yale University School of Medicine, New Haven, CT 06520, USA; b NRG Oncology Statistical and Data Management Center, Roswell Park Comprehensive Cancer Center, Buffalo, NY 14263, USA; c Medical Oncology, Women & Infants Hospital, 101 Dudley Street, Providence, RI 02905, USA; d Gynecologic Oncology, 980 Johnson Ferry Road #910, Atlanta, GA 30342, USA; e Department of Obstetrics & Gynecology, University of Oklahoma, The Stephenson Cancer Center, 800 NE 10th Street, Suite 2500, Oklahoma City, OK 73104, USA; f Gynecologic Oncology, University of New Mexico, 1201 Camino de Salud, Albuquerque, NM 87102, USA; g Department of Obstetrics & Gynecology, Northwestern Medicine Regional Medical Group, 4405 Weaver Parkway, Warrenville, IL 60555-3269, USA; h NRG Oncology, Operations Center-Philadelphia East, Philadelphia, PA, USA; i Division of Gynecologic Oncology, Department of Obstetrics and Gynecology, The Ohio State University Wexner Medical Center, Columbus, OH, USA; j Division of Hematology/Oncology, O’Neal Cancer Center University of Alabama, 176F 10390, 619 19th Street S, Birmingham, AL, USA; k Memorial Sloan Kettering Cancer Center and Weill Cornell Medical College, New York, NY, USA

The authors regret that the authorship of this article was published incorrectly. The above author details are correct, and the online version has been updated.

The authors would like to apologise for any inconvenience caused.

